# Behavior of Colloidal Nanosilica in an Ultrahigh Performance Concrete Environment Using Dynamic Light Scattering

**DOI:** 10.3390/ma12121976

**Published:** 2019-06-19

**Authors:** Douglas Hendrix, Jessica McKeon, Kay Wille

**Affiliations:** 1 Department of Materials Science and Engineering, University of Connecticut, Storrs, CT 06269, USA; douglas.hendrix@uconn.edu; 2 Department of Chemistry, University of Connecticut, Storrs, CT 06269, USA; jessica.mckeon@uconn.edu; 3 Department of Civil and Environmental Engineering, University of Connecticut, Storrs, CT 06269, USA

**Keywords:** ultrahigh-performance concrete, nanosilica, dynamic light scattering, zeta potential, pore solution

## Abstract

The dispersion quality of nanosilica (NS) is an essential parameter to influence and control the material characteristics of nanosilica-enhanced concrete. In this research, the dispersion quality of colloidal nanosilica in simulated concrete environments was investigated using dynamic light scattering. A concrete environment was simulated by creating a synthetic pore solution that mimicked the ionic concentration and pH value of ultrahigh-performance concrete in the fluid state. Four colloidal nanosilica samples were used, ranging in particle sizes from 5 to 75 nm, with differing solid contents and stabilizing ions. It was found that the sodium stabilized 20 nm NS sol remains dispersed at a solid concentration of 2 wt % through a variety of pH values with the inclusion of potassium ions. Calcium ions are a major contributor to the agglomeration of NS sols and only small concentrations of calcium ions can drastically affect the dispersion quality.

## 1. Introduction

**Figure 1:**
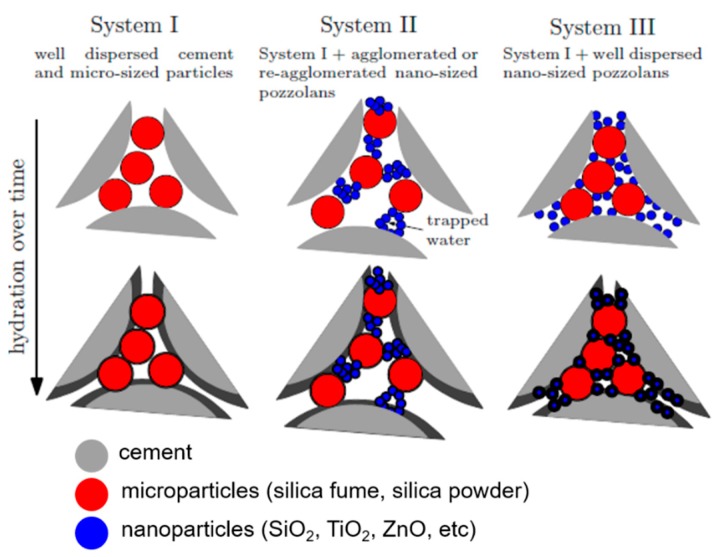
Illustrating the effects of poorly dispersed and well dispersed nanosilica (NS) in a cement system.

The development of concrete research has accelerated in the areas of mechanical and durability properties due to potential benefits of using nanoparticles in concrete mixture design, including nanosilica (NS), nanosized TiO_2_, carbon nanotubes, and graphene oxide [1,2,3,4,5,6]. NS has been widely used due to its small particle size, spherical particle shape, and pozzolanic reactivity which are promising properties to further densify the microstructure of concrete [7,8,9,10]. In previous research, parameters of interest included the type of NS, the size of the particles or the surface area, the concentration of NS, addition or replacement of cement, and water to cement ratios [11,12,13,14,15,16,17]. One of the key challenges in densifying and, therefore, strengthening the microstructure lies in the dispersion quality of the nanoparticles during the mixing process. Figure 1 demonstrates this concept through three systems. System I is a well-dispersed cementitious system with cement and microsized silica, representative of ultrahigh-performance concrete, exhibiting a high particle-packing density and thus enhanced mechanical and durability properties in comparison to conventional concrete [18]. The addition of nanoparticles is a logical step to further increase the particle-packing density. System II highlights what commonly occurs with the inclusion of nanoparticles. These nanoparticles rapidly and irreversibly agglomerate, resulting in an undesired reduction of particle-packing density and thus leading to an inconsistency of mechanical properties. System III is the motivation for this research: Increasing particle-packing density through uniform dispersion of nanoparticles. The control of the dispersion quality will control the concrete’s microstructure and thus will have a direct effect on the material’s mechanical and durability performance. 

The use of NS in concrete has been proven to be a controversial topic due to significant variations of experimental results. Different researchers have reported that nanosilica increases, decreases, and has no effect on the mechanical properties of conventional concrete [19,20,21] and ultrahigh-performance concrete (UHPC) [22,23,24]. UHPC is a cementitious composite characterized by a compressive strength in excess of 150 MPa and enhanced ductility and durability properties as compared to conventional concrete. UHPC contains several constituents of varying sizes including sand, cement, silica powder, and silica fume, decreasing in size respectively. These different size gradations are essential to obtain dense particle-packing and favorable mechanical properties. [25,26,27]. With UHPC containing nanoparticles, the reason for discrepancies in mechanical properties is hypothesized to be a result of the variation in the dispersion quality of NS throughout the matrix. While high dispersion quality is expected to lead to enhanced material properties, poor dispersion quality as illustrated in Figure 1 (System II) compromises the improvement of mechanical properties [28,29]. 

NS particles are susceptible to form large agglomerates due to their very high specific surface area (50–750 m^2^/g). Colloidal NS, as opposed to a dry powder, provides NS particles in a well-dispersed state, free of significant agglomeration. These discrete particles have hydroxylated surfaces and achieve stability by possessing a net negative charge on the surface, strong enough to repulse the van der Waals attractive forces. If different ions are introduced to the sol, this surface charge can be altered and can lead to agglomeration and gelation of the NS [30,31,32,33,34]. Therefore, adding well-dispersed NS to the concrete mixture can lead to agglomeration and gelation resulting from the release of ions during cement hydration. 

Understanding the dispersion mechanisms of NS is essential in the engineering and design of novel nanomaterial enhanced cementitious composites. Besides enhanced packing density, improved pore structure, and thus enhanced mechanical and durability properties, these new composites can exhibit specific functionality by inheriting properties of the added nanoparticles, such as air depolluting, elasticity control, and acceleration of hydration kinetics [35,36,37,38].

The goal of this research is to understand the mechanisms that cause NS destabilization and agglomeration in UHPC. It is hypothesized that even well-dispersed colloidal NS could result in agglomerates in the concrete environment of UHPC. The challenge is to define and investigate the dispersion quality of colloidal NS sols under concrete mixing conditions. In this paper, particle size distribution (PSD) through dynamic light scattering (DLS) and zeta potential (ZP) were measured and analyzed to evaluate the dispersion quality. NS sols were diluted, pH was altered, and added to a synthetic pore solution to further understand NS destabilization.

### 1.1. Experimental Theory

#### 1.1.1. Colloidal NS

Most commercially-available NS sols are stabilized at a pH range of 7–10. Silica particles are negatively charged as a result of the silanol groups (Si–O–H) on the surface. This provides a repulsive force between adjacent particles. Since the silica must be electrically neutral, counter ions such as potassium, sodium, and ammonia are inserted into the solution. These positively charged ions balance the charge by being attracted to the negatively charged surface of the silica particles [39]. NS sols can become destabilized for various reasons and once the van der Waals attractive forces are greater than the repulsive forces, the particles irreversibly agglomerate [40]. Silica can also be stabilized at low pH by removing all ions, resulting in slightly negatively charged particles, as this is the case with NS-20a used in this study.

The Derjaguin-Landau-Verwey-Overbeek (DLVO) theory predicts the stability of charged particles in a liquid medium by factoring in van der Waals attractive forces and electrostatic repulsion. DLVO theory predicts that if the electrostatic forces are greater than the van der Waals forces, the colloid will not agglomerate. DLVO provides a fundamental theory of the stability of particles in different pH and different ionic concentrations. However, in experimental practice with silica, DLVO breaks down at low pH. DLVO predicts at the isoelectric point at a pH of 2, the system should lack stability. However, at or near this point, colloidal silica has demonstrated stability [40]. This indicates that there are forces not considered by DLVO that significantly affect the stability, such as the steric hindrance effect. These properties of silica have made it difficult to predict stability, especially with the use of multivalent ions, varying particle sizes, and polymers. Therefore, experimental studies are necessary.

The pH of NS sols can affect the stability of the sol. At high pH (pH > 11), silica starts to dissolve, forming silicate ions, leading to destabilization [41]. At low pH, the repulsive forces are suppressed, resulting in a lower stability. Another potential source for NS destabilization is the ionic composition and concentration in the medium surrounding NS particles [42]. Monovalent and divalent ions, such as potassium (K^+^) and calcium (Ca^2+^) used in this study, alter the ionic charge of sols. This results in two mechanisms of agglomeration. First, the adsorption of potassium and calcium ions can lead to a reduction in the electrical double layer (further discussed in Section 1.1.3.). This reduces the electrostatic repulsive forces and attractive forces dominate. In addition to compressing the electrical double layer, these ions can form bridges between NS particles. A positively charged ion can neutralize a site on the silica surface. If two of these uncharged surfaces collide, the ion can coordinate with the oxygens of the silanol groups and the surface bonded water, forming a physical bridge between two silica particles [41].

For example, at a pH between 6 and 9, alkali metals at a high enough concentration can result in agglomeration. However, at a higher pH (pH > 10), it has been observed that potassium, rubidium, and cesium do not cause agglomeration at high concentrations. It is hypothesized that these ions form a complete double layer around the particle effectively shielding the silica surfaces from coming into contact [41]. Why this happens at a higher pH and not a lower pH is unknown, again supporting the observation that DLVO theory is not perfect.

These two effects of agglomeration are amplified with divalent ions such as calcium. A divalent ion neutralizes one negative surface charge and thus releases a hydrogen ion, while the originally divalent ion retains a positive charge. Since the ion still retains a positive charge, it can neutralize an additional site on an adjacent silica particle, forming a physical bridge between particles. The adsorption of calcium results in a mosaic of positive and negative charges on the surface which facilitates the agglomeration of particles as there are many sites for opposite charges to attract each other, as proposed by Goodman [43] and Iler [44]. When there are two different ions adsorbed onto the surface, such as potassium and calcium, coagulation can occur more rapidly. The addition of potassium reduces the overall repulsive forces by neutralizing some of the surface charge. Calcium then causes bridging and coagulation at lower concentrations compared to sols without salt [45,46].

#### 1.1.2. Dynamic Light Scattering

DLS was used to determine the PSD of colloidal NS particles or their agglomerates. DLS measures time-dependent fluctuations in the intensity of scattered light of particles in a suspension which move in a random Brownian motion [47,48]. These fluctuations are processed into an autocorrelation function and applied to fitting algorithms [49]. In this research, the cumulant method [50] has been used. It is the most common method to obtain a particle size distribution from the autocorrelation function.

Two measurements from DLS are primarily used in this research: Size (z-average) and polydispersity index (PDI). The z-average is the intensity-weighted mean hydrodynamic size of the particles, which is derived from the cumulants analysis. The PDI is a dimensionless number calculated from a fit to the cumulant data. It is a measure of how narrow or broad the PSD is. Smaller values indicate a narrow size distribution. Values greater than 0.5 indicate a very broad distribution and results become more qualitative than quantitative.

In this research, a lower z-average, closer to the manufacturers supplied value, indicates a better dispersion quality. A higher z-average indicates a lower dispersion quality, as agglomeration has occurred. In addition, lower PDI values are preferred, indicating a small range of particle sizes. 

#### 1.1.3. Zeta Potential

Obtaining the zeta potential (ZP), or the electrokinetic potential, is a technique to quantify potential stability of suspended particles. By measuring the electrophoretic mobility of a particle, the ZP is calculated. A higher magnitude of ZP corresponds to a higher degree of electrostatic repulsion between the charged particles and, thus, a higher dispersion stability, or stronger agglomeration resistance. As a general rule of thumb, magnitudes greater than 30 mV indicate stability and magnitudes less than 30 mV indicate higher potential for agglomeration and coagulation [47,51].

The distribution of ions in a solution is affected by charged particles. These charged particles result in a higher concentration of ions close to the surface. In this region, known as the Stern layer, ions such as Na^+^ and K^+^ are strongly bound. An outer layer, known as the diffuse layer, contains ions that are less strongly bound. These two layers form an electrical double layer around each particle. These two layers extend a finite distance from a particle surface and are influenced by several factors including pH, ionic concentration and composition. The potential at the edge of electrical double layer with the surrounding environment is the ZP [40,47,52]. The thickness of the diffuse layer is the Debye length. The Debye length is a measure of how far the electrostatic forces persist from the particle surface. A larger Debye length keeps particles further apart which increases sol stability. In aqueous solutions of monovalent ions, the length is reciprocally proportional to the square root of the ionic concentration. In a 0.1 M solution (the maximum concentration of KOH in this work) the Debye length is 0.96 nm. In a 0.4 M solution of divalent ions, the Debye length is 0.28 nm [51]. This is a very short distance, which is one reason why NS can be difficult to stabilize in some environments.

## 2. Materials and Methods

### 2.1. Experimental Parameters

#### 2.1.1. Colloidal NS

**Figure 2:**
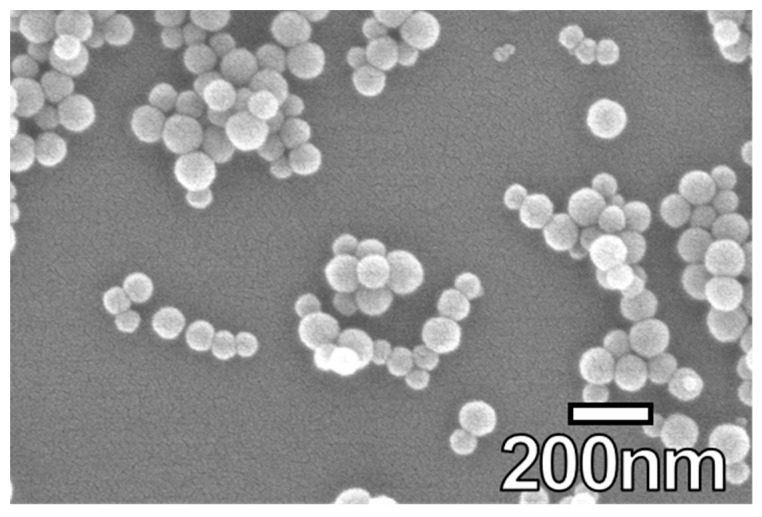
Scanning electron microscopy (SEM) image of NS-75.

Four commercially available colloidal NS sols were used in this study. The compositions and average physical properties provided by the manufacturer of the NS sols are shown in Table 1. Figure 2 shows their spherical shape.

#### 2.1.2. Dynamic Light Scattering

The PSD of NS was obtained by DLS using a Zetasizer Nano ZS (Malvern Instruments Ltd., Malvern, UK). Measurements were performed using disposable polystyrene cuvettes at room temperature (25.0 °C). The dispersant (water) properties were set to default values of viscosity: 0.8872 cP and refractive index: 1.330. The material absorption coefficient was set to 0.001 and the refractive index to 1.50 [53]. These values were kept constant throughout all measurements. Samples were thermally equilibrated for 30 s and the angle of detection was set to 173° (Non-Invasive Backscatter (NIBS)). Three measurements were performed for each sample at 11 runs per measurement, 10 seconds per run. The measurements reported in the results section are the mean of the corresponding three measurements. The general purpose algorithm and the method of cumulants were used for analysis. All measurements were taken within one hour of sample preparation. Raw materials were stored at room temperature.

#### 2.1.3. Zeta Potential

ZP was measured using the Zetasizer Nano ZS with the dip cell accessory. The same material parameters were used as in the particle size measurements. Four measurements were performed for each sample at 30 runs per measurement. The voltage selection was set to automatic, with values between 1 V and 20 V. ZP measurements were taken from the same sample as the corresponding DLS measurement.

#### 2.1.4. Sample Preparation

**Figure 3:**
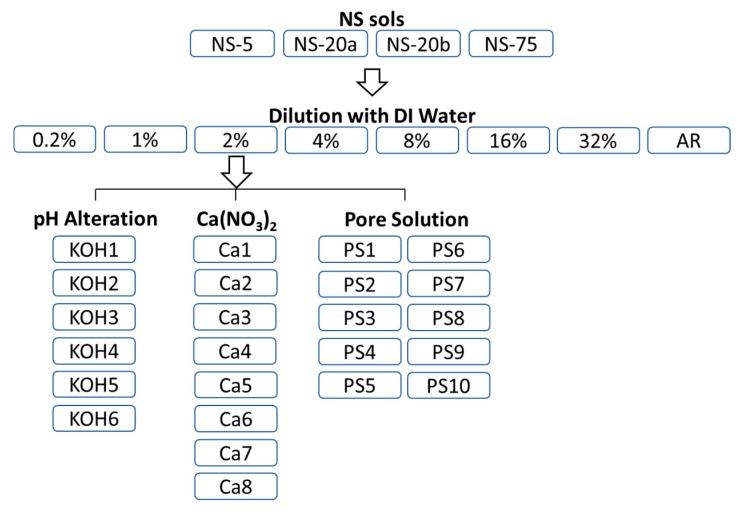
Experimental design DI: Deionized. AR: As received.

The parameters for testing the four NS sols from Table 1 included varying solid concentration by adding deionized (DI) water, varying pH value by adding 0.1 M aqueous potassium hydroxide (KOH) or 0.1 M hydrochloric acid (HCl), varying amounts of calcium nitrate (Ca(NO_3_)_2_·4H_2_O), and adding UHPC-like synthetic pore solution (PS) as outlined by Schrofl et al. (9.72 g Ca(NO_3_)_2_·4H_2_O dissolved in 148.5 g of 0.1 mol/L aqueous KOH solution) [54]. An overview of the test matrix is shown in Figure 3 highlighting the four test parameters. The concentration of NS in Figure 3 is by weight percent. It was observed that PS causes irreversible agglomeration. The two ingredients of the PS were isolated to further understand the system.

NS sols at 2% solid content were chosen to investigate the effect of pH value, concentration of Ca(NO_3_)_2_, and ionic concentration of the PS. The alterations and concentrations are summarized in Table 2. The synthetic PS contained 0.1 M of KOH and 0.4 M of Ca(NO_3_)_2_·4H_2_O in accordance to Reference [54]. KOH6, Ca8, and PS10 correspond to the molar concentration of the as received synthetic PS. Each successive concentration step down was half of the prior concentration. The pH value of the samples was measured using pH paper in increments of 0.5. DI water, aqueous KOH, aqueous Ca(NO_3_)_2_, and pore solution were filtered to 0.1 µm using Anotop (GE Whatman, Marlborough, MA, USA) syringe filters before mixing with NS.

#### 2.1.5. CryoSEM

Cryogenic scanning electron microscopy (cryoSEM) was used to visualize the NS particle dispersion after mixing with cement paste to be able to determine the technique’s efficacy for samples of this type. Cement paste was dropped on a 2 mm rivet holder and shock frozen by immersing into a liquid nitrogen slush. Afterwards, samples were fractured, etched, and sputter-coated with Au/Pt in a Leica EM MED020 with QSG10 (Leica Microsystems Inc., Buffalo Grove, IL, USA). Samples were then transferred using the Leica EM VCT100 (Leica, Wetzlar, Germany) and imaged with an FEI Nova NanoSEM 450 (FEI, Hillsboro, OR, USA). The accelerating voltage was 10 kV and the working distance was 5.5 mm.

A cement paste sample was mixed using white Portland cement (w/c ratio 0.3) (Type I, ASTM C150, Lehigh White Cement Co., Waco, TX, USA) and NS-75 (1.5% bwoc) [26]. The cement paste was mixed in a LabRAM Mixer (Resodyn Acoustic Mixers, Butte, MT, USA) at an intensity of 50% for a total mixing time of 300 s. The time between initial hydration and freezing was 1 h.

## 3. Results

### 3.1. Diluting NS

**Figure 4:**
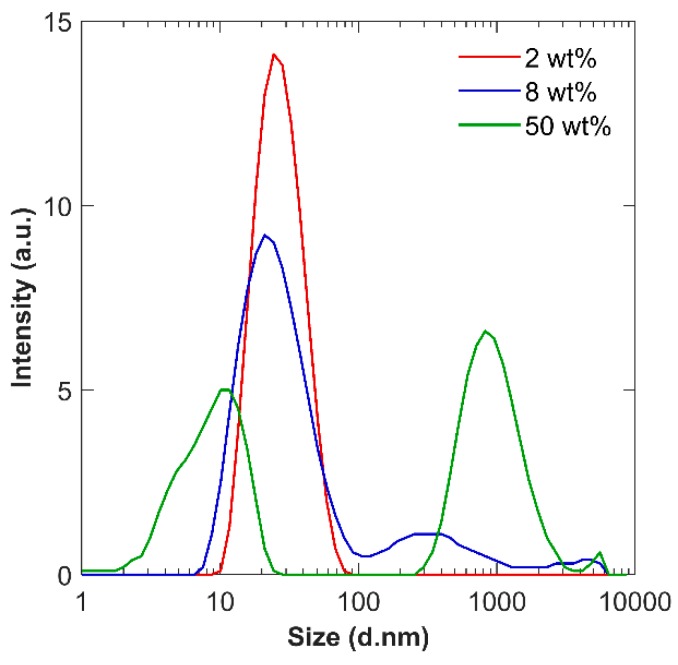
Selected particle size distribution (PSD) of dilution of NS-20b demonstrating the transition from bimodal to monomodal distributions.

All four NS sols were altered and measured for particle size distribution (PSD) and zeta potential (ZP). The effect of the concentration of NS particles was first studied to isolate the potential influence of the number of particles in a sol. All sols except NS-75 in their as-received state were found to be polydisperse, with a PDI greater than 0.5. Interestingly, the PSD for NS-20b changed as it was diluted. It exhibited a bimodal distribution at higher concentrations and at concentrations below 8 wt % solid, it exhibited a monomodal distribution, as shown in Figure 4, indicating a better dispersion quality. At 2 wt %, the z-average was 24.7 nm (standard deviation (SD) = 0.4), which is close to the provided value of 20 nm by the manufacturer. This suggests that at higher concentrations, the particles tend to weakly agglomerate. By reducing the concentration, there is a greater time and distance between particle collisions, resulting in a better dispersion quality.

NS-20a was too polydisperse for DLS until it was diluted to a concentration of 0.2 wt %. The z-average was 68.3 nm (SD = 1.5), about three times the supplied value. NS-5 was too polydisperse for quantitative results throughout all concentrations. It is hypothesized that these two sols were polydisperse because of the increased specific surface area of the smaller particle diameters. The increased surface energy was not strongly influenced by the reduction in concentration. The complete results are reported in the Supplementary Materials. 

**Figure 5:**
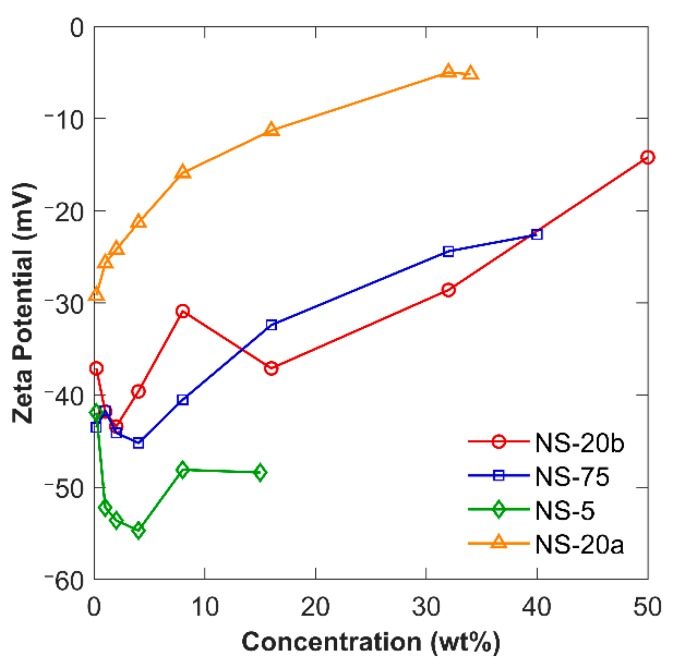
Zeta potential of NS sols through a range of dilution.

The ZP as a function of solid concentration is shown in Figure 5. In the cases of the two sodium-stabilized NS sols, NS-20b and NS-75, the size and ZP trends were directly correlated. As the size decreased, indicating a better dispersion quality, the ZP increased, indicating a higher stability. On this plot, a lower, more negative value indicates a greater ZP. At a concentration between 2% and 4%, the sols experienced the largest ZP, indicating the highest stability. 

Based on the experimental results, there exists an ideal solid concentration for the greatest stability in DI water. Based on the PSD and ZP measurements, this can be concluded to be about 2 wt % solid NS. This conclusion can only be made for NS-20b and NS-75, as the two other sols were not suitable for DLS due to their high polydispersity and produced inconclusive results. 

### 3.2. pH Adjustment

The pH of the NS sols was adjusted from about 2 to 12 at 2 wt % solid using HCl and KOH. Throughout pH adjustment, the average size of the sols was remarkably consistent. For the two sodium-stabilized sols, the difference between the maximum and minimum particle size was 7.5 nm (NS-20b) and 7.4 nm (NS-75). For the ammonia-stabilized NS-5, the difference was 5.4 nm. The non-stabilized NS-20a experienced a difference in size of 8.9 nm.

**Figure 6:**
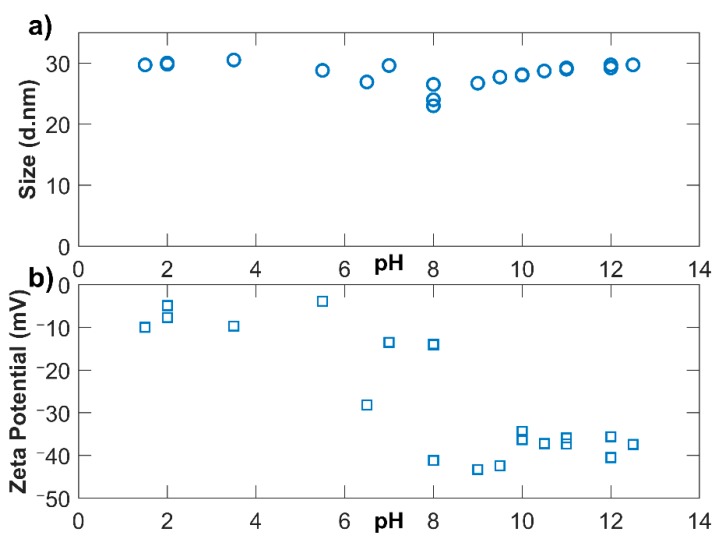
pH adjustment of NS-20b with 0.1 M HCl and 0.1 M KOH; (**a**) displays z-average while (**b**) reports zeta potential.

NS-20b had its minimum size at a pH of 8: 23.0 nm (SD = 0.4). The maximum magnitude of ZP was at a pH of 9: −43.3 mV (SD = 3.3). It is not surprising this was observed near its supplied pH value of 9 since the sol is optimized by the manufacturer to experience the greatest stability at this pH. From Figure 6 it is apparent that the average size reaches its greatest value at the ends of the pH scale. The other three sols also generally experienced the largest sizes at the ends of the pH spectrum.

However, the ZP trends were not as apparent. Qualitatively, the ZP was lower at lower pH and higher at higher pH, as is loosely observed in Figure 6. One reason is because the HCl used to lower the pH compressed the electrical double layer. Compared to KOH, the HCl had a negative effect on the ZP. For NS-20b, a reduction in ZP was measured but agglomeration did not occur as would be expected from DLVO theory, exhibited in Figure 6. Similar trends were seen for NS-5 and NS-75. The complete data is presented in the Supplementary Materials.

In a similar experiment, KOH was also added in concentrations relative to the PS to isolate its effect. At the two lowest concentration, KOH1 and KOH2, NS-20b exhibited some agglomeration on the order of magnitude of 3000 nm, while at higher concentrations it experienced a better dispersion quality. NS-20a had a polydisperse size distribution at 2% solid, however, with the introduction of KOH, the dispersion changed to monodisperse distribution. It reached an agglomeration state of about 40 nm, or about twice the manufacturers supplied value, which remained fairly consistent during significant pH adjustment. The addition of KOH altered the electrical double layer and resulted in a metastable state, where slight agglomeration occurred to yield the average size of 40 nm. ZP measurements of NS-20a experienced a similar trend, remaining at about −32 mV. NS-5 exhibited a similar trend of reaching a metastable agglomeration state, although it took an increased amount of KOH to reach a monodisperse distribution. The initial agglomeration state was about twice the supplied manufacturer’s value and approached three times that value at the highest concentration of KOH. 

Above pH 11, silica was expected to start dissolving. Effects of dissolution were not apparent. Several samples were observed with DLS over a period of 96 h at a pH above 12. The average particle size remained consistent, indicating that the particles were not dissolving. The remarkable stability of the NS sols through a wide range of pH adjustment and over periods of time at high pH suggests that the manufacturer’s proprietary stabilization method is fairly robust, which should be useful for achieving uniform dispersion in a concrete mix.

### 3.3. Calcium Nitrate

Calcium nitrate was added in several concentrations to isolate its effect in the PS at 2 wt % solid. For NS-20b and NS-5, the sols were able to tolerate the addition of calcium nitrate up to Ca4. Above Ca4, there was significant agglomeration and DLS could not provide meaningful data. The other two sols, NS-75 and NS-20a, had a fairly constant size throughout the range of Ca^2+^ concentrations. From the average size alone, it is apparent that Ca(NO_3_)_2_ has a greater influence on agglomeration than KOH. After 24 h, all NS-Ca(NO_3_)_2_ samples were observed to sediment to the bottom of the cuvette, indicating coagulation had occurred. It was hypothesized that the particle size and stabilizing ion would have an influence on the agglomeration with the addition of Ca^2+^. However, no conclusions could be drawn based on the PSD.

ZP measurements provide greater insight into the stability of these samples. All four sols experienced a reduction in the magnitude of the ZP and switched from an initially negative ZP to a positive ZP. NS-75 at Ca8 had a ZP of 29.2 mV (SD = 12.3), a difference of almost 78 mV without Ca(NO_3_)_2_. Therefore, it can be concluded that Ca^2+^ significantly changes the surface charge of the silica particles. This happens more rapidly than the K^+^ ions due to the higher valency of Ca^2+^. These ions offset the surface charge and reduce the electrostatic repulsive forces. The bridging of particles with calcium is hypothesized to be the main cause of agglomeration. Small concentrations (<Ca2) of Ca(NO_3_)_2_ resulted in large changes to the ZP. These results were consistent to those of Iler [41] and the hypothesized mechanisms of agglomeration can be reported with confidence. These findings illustrate the importance of DLS and ZP which provide different information on the same sample.

### 3.4. Synthetic Pore Solution

Synthetic PS was added in several concentrations to study the effects of the two previous parameters combined. The concentration of the PS was reduced by dilution with DI water. Again, this was added to the 2% solid.

NS-20b and NS-75 both experienced agglomeration beginning at PS4, with significant agglomeration above that concentration. When KOH and Ca(NO_3_)_2_ were combined to create the synthetic PS, its propensity to cause agglomeration was more significant than either component alone, as predicted by Plank et al. [55]. The effects of increasing pH and the introduction of two cations, K^+^ and Ca^2+^, accelerated the agglomeration. The magnitude of the ZP was greatly reduced and the reduction occurred quicker with PS compared to either component alone. It is interesting to note that in no case was the ZP positive, as was observed with the addition of Ca^2+^ only. Before ZP could approach 0, significant agglomeration occurred. These results were expected from the hypotheses of Iler [41] and Tadros [46] and can report these mechanisms with confidence. 

**Figure 7:**
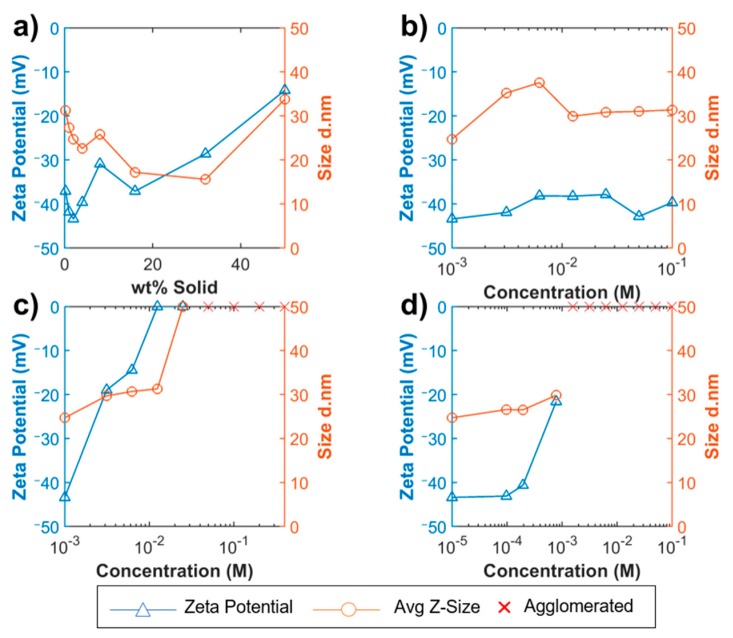
ZP and size measurements for NS-20b: (**a**) solid concentration varied with DI water; (**b**) potassium hydroxide concentration varied with DI water; (**c**) calcium nitrate concentration varied with DI water; (**d**) pore solution concentration varied with DI water.

PS10 is the pore solution concentration that would be equivalent in pH and ionic concentration to an actual UHPC environment. It is apparent from Figure 7d and Table S13 that NS stability in an actual concrete environment is far from being achieved. Since this pore solution only contains water, KOH, and Ca(NO_3_)_2_ it can be inferred that the Ca(NO_3_)_2_ is the main contributor to NS agglomeration, due to its bridging effect between silica particles.

### 3.5. NS Visualization

**Figure 8:**
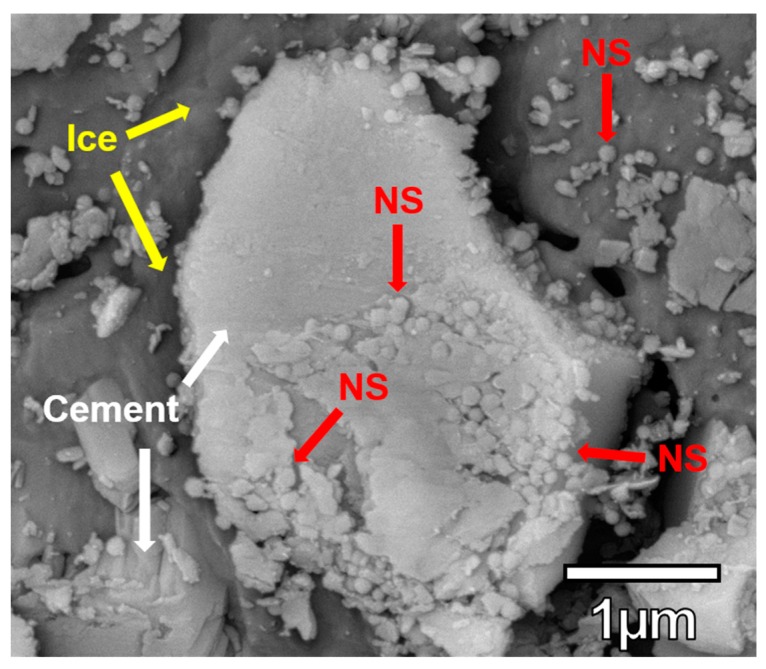
CryoSEM image showing unreacted NS on a cement grain.

An important aspect of understanding dispersion and agglomeration of NS in a true cementitious environment is to directly observe what happens in a concrete sample. This can often be challenging as NS can lead to an increased amount of C-S-H gel and therefore making individual NS particles indistinguishable. Observation of NS was explored by using cryoSEM, as shown in Figure 8. Since the hydration was stopped after 1 hour, some NS was unreacted and visible. No NS particles were observed to be completely separate from another NS particle and the NS appears to be spread out over about half the cement grain. This data was not used quantitatively but proved that this will be a viable method to explore the effects of cementitious pore solution on the dispersion of NS. Future data analysis could include calculating the number of particles per unit area to quantify the dispersion quality. Based on direct observation, the effects of superplasticizers on the dispersion of cement, silica fume, and NS can be investigated. Ongoing research will utilize cryoSEM to visualize the effects of superplasticizers tailored specifically for improved dispersion of NS. 

## 4. Conclusions

In this research, the stability of NS in a concrete environment has been studied. The optimal concentration for the best dispersion quality of colloidal NS in a cementitious environment is 2 wt % solid. This concentration allows for sufficient screening of short-range van der Waals attractive forces. The supplied NS sols are fairly pH-robust since they tolerate a wide range of pH changes without agglomerating. This is especially the case with the addition of KOH. The magnitude of the zeta potential remained high and this helped to improve the dispersion quality. On the contrary, Ca^2+^ ions are the main contributor to the agglomeration of NS in pore solution. Its higher valency further compresses the electrical double layer, causing the ZP to shift and thus results in a positive surface charge. This neutralization of negative sites results in a mosaic of positive and negative surface charges, leading to bridging between silica particles. By analyzing the size and ZP measurements, NS-20b experienced the best dispersion quality compared to the other three sols. This is due to its intermediate surface area, compared to higher (NS-5) and lower (NS-75) surface areas. The smaller particles were more susceptible to agglomeration due to the higher surface energy. Achieving stability in full strength PS, representing UHPC, is far from being accomplished. NS sols of similar stabilization methods and size will be of greater focus for future studies to obtain stability in PS. Methods to neutralize the effects of Ca^2+^ will be specifically targeted to improve the dispersion quality in the presence of PS. Finally, cryoSEM proved to be a valuable tool for cementitious material characterization in the fluid state. The ability to stop hydration and image the microstructure in the fluid state will be critical to understanding the dynamics of NS interaction, and thus their dispersion quality in a cementitious environment.

## Figures and Tables

**Table 1:** Properties of NS sols.

Property	NS-5	NS-20a	NS-20b	NS-75
Particle Size (nm)	5	20	20	75
Surface Area (m^2^/g)	600	150	150	40
% SiO_2_	15	34	50	40
pH	9.0	2.8	9.0	8.4
Specific Gravity	1.09	1.23	1.39	1.29
Viscosity (cP)	<10	<10	55	10
Stabilizing Ion	Ammonium	—	Sodium	Sodium
% Na_2_O	0.02	0.04	0.40	0.30

**Table 2:** Test specifications for NS alteration.

2% Solid NS					
pH Alteration	Molarity (mol/L)	Ca(NO_3_)_2_	Molarity (mol/L)	Pore Solution	Molarity of KOH (mol/L)
KOH0	0	Ca0	0	PS0	0
				PS1	9.77 × 10^−5^
				PS2	1.95 × 10^−4^
		Ca1	3.13 × 10^−3^	PS3	7.81 × 10^−4^
		Ca2	6.25 × 10^−3^	PS4	1.56 × 10^−3^
KOH1	3.13 × 10^−3^	Ca3	1.25 × 10^−2^	PS5	3.13 × 10^−3^
KOH2	6.25 × 10^−3^	Ca4	2.50 × 10^−2^	PS6	6.25 × 10^−3^
KOH3	1.25 × 10^−3^	Ca5	5.00 × 10^−2^	PS7	1.25 × 10^−2^
KOH4	2.50 × 10^−3^	Ca6	1.00 × 10^−1^	PS8	2.50 × 10^−2^
KOH5	5.00 × 10^−3^	Ca7	2.00 × 10^−1^	PS9	5.00 × 10^−2^
KOH6	1.00 × 10^−3^	Ca8	4.00 × 10^−1^	PS10	1.00 × 10^−1^

## Supplementary Materials

The following material is available online at https://www.mdpi.com/1996-1944/12/12/1976/s1.
