# Supplementary material for: Behavior of Colloidal Nanosilica in an Ultrahigh Performance Concrete Environment Using Dynamic Light Scattering
Source: Materials (Basel). 2019 Jun 19;12(12):1976. doi: 10.3390/ma12121976 (PMC6631289; doi:10.3390/ma12121976)
Supplement: Supplementary file 1 [file materials-12-01976-s001.pdf]

Supplementary Materials: Behavior of Colloidal Nanosilica in an Ultra-High Performance Concrete Environment Using Dynamic Light Scattering

Douglas Hendrix [1], Jessica McKeon [2] and Kay Wille [3,*]

Table S1. Dilution of NS-20b.

% Solid	pH	Size (d.nm)	SD	PdI	SD	ZP (mV)	SD
0.2	7	31.2	0.3	0.13	0.01	−37.1	1.1
1	7	27.3	0.5	0.14	0.00	−41.8	1.9
2	7.5	24.7	0.4	0.14	0.00	−43.4	0.7
4	8	22.6	0.3	0.17	0.00	−39.6	1.5
8*	8.5	25.8	0.4	0.35	0.01	−30.9	2.5
16*	9	17.2	0.4	0.28	0.02	−37.1	1.5
32*	9.5	15.6	0.4	0.44	0.01	−28.6	1.4
50*	9.5	33.8	6.7	0.74	0.04	−14.2	1.4

Note: * indicates a bimodal distribution.

Table S2. Dilution of NS-75.

% Solid	pH	Size (d.nm)	SD	PdI	SD	ZP (mV)	SD
0.2	7	95.9	1.0	0.03	0.02	−43.5	1.5
1	7	97.8	0.4	0.06	0.03	−41.7	1.8
2	7	96.2	1.1	0.07	0.00	−44.1	1.3
4	7.5	92.7	1.3	0.08	0.02	−45.2	1.3
8	7.5	89.8	0.7	0.10	0.00	−40.5	2.8
16	8	82.5	1.1	0.11	0.02	−32.4	1.1
32	8.5	71.5	0.1	0.19	0.00	−24.4	1.0
40	8.5	70.9	0.7	0.26	0.01	−22.6	2.2

Table S3. Dilution of NS-5. Values that could not be obtained with DLS are omitted.

% Solid	pH	Size (d.nm)	SD	PdI	SD	ZP (mV)	SD
0.2	7	-	-	>0.5	-	−41.9	4.3
1	8	-	-	>0.5	-	−52.2	0.3
2	8.5	-	-	>0.5	-	−53.6	2.3
4	9	-	-	>0.5	-	−54.7	0.9
8	9	-	-	>0.5	-	−48.1	2.9
15	8.5	-	-	>0.5	-	−48.4	1.5

Table S4. Dilution of NS-20a. Values that could not be obtained with DLS are omitted.

% Solid	pH	Size (d.nm)	SD	PdI	SD	ZP (mV)	SD
0.2*	6	68.3	1.5	0.24	0.01	−29.2	1.1
1*	6	64.1	0.5	0.57	0.00	−25.7	0.9
2	5	-	-	>0.7	-	−24.2	1.1
4	5.5	-	-	>0.7	-	−21.3	1.1
8	5.5	-	-	>0.7	-	−15.9	0.8
16	5	-	-	>0.7	-	−11.3	0.2
32	4	-	-	>0.7	-	−5.0	0.2
34	4	-	-	>0.7	-	−5.2	0.5

Note: * indicates a bimodal distribution.

Table S5. KOH addition to NS-20b.

Sample	pH	Size (d.nm)	SD	PdI	SD	ZP (mV)	SD
KOH0	7.5	24.7	0.4	0.14	0.00	−43.4	0.7
KOH1	8.5	35.2	0.4	0.26	0.01	−41.9	1.2
KOH2	9.5	37.6	0.3	0.24	0.01	−38.2	0.7
KOH3	10	29.9	0.4	0.09	0.01	−38.2	1.3
KOH4	10.5	30.8	0.4	0.07	0.01	−37.9	2.1
KOH5	11.5	31.0	0.1	0.07	0.02	−42.8	0.7
KOH6	12	31.4	0.2	0.05	0.00	−39.7	1.0

Table S6. KOH addition to NS-75.

Sample	pH	Size (d.nm)	SD	PdI	SD	ZP (mV)	SD
KOH0	7	96.2	1.1	0.07	0.00	−44.1	1.3
KOH1	8.5	93.0	0.2	0.06	0.01	−50.7	1.0
KOH2	9.5	94.2	1.2	0.03	0.01	−50.5	0.6
KOH3	9.5	93.2	0.7	0.03	0.02	−54.4	3.8
KOH4	9.5	92.4	1.3	0.04	0.01	−56.7	1.6
KOH5	10.5	93.5	2.2	0.02	0.02	−54.5	1.9
KOH6	12.5	94.6	1.3	0.03	0.02	−40.6	1.4

Table S7. KOH addition to NS-5.

Sample	pH	Size (d.nm)	SD	PdI	SD	ZP (mV)	SD
KOH0	8.5	-	-	-	-	−53.6	2.3
KOH1*	9	10.9	0.2	0.43	0.01	−65.5	3.6
KOH2*	9	11.3	0.1	0.42	0.01	−58.9	1.5
KOH3	9.5	10.9	0.1	0.31	0.01	−55.8	2.3
KOH4	10	12.9	0.1	0.23	0.00	−49.7	1.5
KOH5	11	13.9	0.0	0.205	0.003	−49.7	3.7
KOH6	11.5	14.8	0.1	0.21	0.01	−44.1	1.0

Note: * indicates a bimodal distribution.

Table S8. KOH Addition to NS-20a.

Sample	pH	Size (d.nm)	SD	PdI	SD	ZP (mV)	SD
KOH0	4	-	-	> 0.7	-	−24.2	1.1
KOH1	8.5	41.9	1.8	0.27	0.01	−30.7	0.5
KOH2	9	40.3	1.7	0.24	0.00	−32.5	0.4
KOH3	10	39.3	1.5	0.18	0.01	−32.6	1.6
KOH4	11	39.8	1.1	0.17	0.01	−30.5	1.3
KOH5	12	40.5	1.3	0.15	0.01	−30.9	1.7
KOH6	13	40.2	1.1	0.14	0.01	−33.1	1.6

Table S9. $Ca(NO_3)_2$ addition to NS-20b.

Sample	pH	Size (d.nm)	SD	PdI	SD	ZP (mV)	SD
Ca0	7.5	24.7	0.4	0.14	0.00	−43.4	0.7
Ca1	7	29.7	0.2	0.08	0.01	−19.0	0.5
Ca2	7	30.7	0.1	0.06	0.02	−14.5	1.4
Ca3	7	31.3	0.2	0.06	0.01	0.2	13.2
Ca4	6	50.7	0.3	0.20	0.01	0.5	11.5
Ca5	7.5	-	-	-	-	-	-
Ca6	8	-	-	-	-	-	-
Ca7	7.5	-	-	-	-	-	-
Ca8	7.5	-	-	-	-	-	-

Table S10. $Ca(NO_3)_2$ addition to NS-75.

Sample	pH	Size (d.nm)	SD	PdI	SD	ZP (mV)	SD
Ca0	7	96.2	1.1	0.07	0.00	−44.1	1.3
Ca1	5	91.3	0.0	0.04	0.03	−11.4	4.4
Ca2	5.5	93.1	0.9	0.05	0.01	−9.6	7.6
Ca3	6	92.1	0.6	0.02	0.02	11.6	3.4
Ca4	5	92.7	0.8	0.02	0.01	9.2	6.5
Ca5	7.5	92.8	0.4	0.03	0.01	16.8	15.3
Ca6	7.5	93.9	1.1	0.02	0.01	10.5	25.2
Ca7	7.5	94.3	1.1	0.02	0.01	21.5	22.3
Ca8	3.5	96.3	0.7	0.04	0.01	29.2	12.3

Table S11. $Ca(NO_3)_2$ addition to NS-5.

Sample	pH	Size (d.nm)	SD	PdI	SD	ZP (mV)	SD
Ca0	8.5	-	-	-	-	−53.6	2.3
Ca1	8.5	11.3	0.3	0.35	0.02	−43.0	0.7
Ca2	8.5	11.9	0.1	0.23	0.01	−34.9	0.9
Ca3	8.5	17.6	0.3	0.18	0.01	−20.3	2.7
Ca4	8.5	-	-	-	-	−8.2	1.2
Ca5	-	-	-	-	-	-	-
Ca6	-	-	-	-	-	-	-
Ca7	-	-	-	-	-	-	-
Ca8	-	-	-	-	-	-	-

Table S12. $Ca(NO_3)_2$ addition to NS-20a.

Sample	pH	Size (d.nm)	SD	PdI	SD	ZP (mV)	SD
Ca0	4	-	-	>0.7	-	−24.2	1.1
Ca1	7	63.0	0.3	0.21	0.01	−5.4	0.7
Ca2	7	65.0	0.2	0.20	0.01	−4.2	0.6
Ca3	6.5	64.4	0.7	0.20	0.01	−2.4	0.9
Ca4	6	69.4	0.7	0.22	0.01	5.1	8.4
Ca5	5.5	66.1	0.3	0.19	0.00	24.3	3.4
Ca6	6	66.6	0.3	0.21	0.01	3.6	12.4
Ca7	3	68.0	0.5	0.20	0.02	29.2	18.5
Ca8	6.5	70.9	0.5	0.20	0.00	-	-

Table S13. Pore solution addition to NS-20b. Values that could not be obtained with DLS were omitted.

Sample	pH	Size (d.nm)	SD	PdI	SD	ZP (mV)	SD
PS0	7.5	24.7	0.4	0.14	0.00	−43.4	0.7
PS1	7.5	26.6	0.6	0.16	0.02	−43.1	0.9
PS2	8	26.5	0.3	0.12	0.01	−40.6	2.3
PS3	8	29.8	0.3	0.08	0.01	−21.6	0.5
PS4	-	-	-	-	-	−2.8	0.5
PS5	-	-	-	-	-	-	-
PS6	-	-	-	-	-	-	-
PS7	-	-	-	-	-	-	-
PS8	-	-	-	-	-	-	-
PS9	-	-	-	-	-	-	-
PS10	-	-	-	-	-	-	-

Table S14. Pore solution addition to NS-75. Values that could not be obtained with DLS were omitted.

Sample	pH	Size (d.nm)	SD	PdI	SD	ZP (mV)	SD
PS0	7	96.2	1.1	0.07	0.00	−44.1	1.3
PS1	7	92.9	0.5	0.03	0.01	−37.1	0.7
PS2	7	93.9	0.3	0.07	0.02	−32.0	2.5
PS3	7.5	93.4	1.3	0.04	0.02	−23.1	1.1
PS4	8	210.7	18.4	0.29	0.02	−20.6	2.8
PS5	-	-	-	-	-	-	-
PS6	-	-	-	-	-	-	-
PS7	-	-	-	-	-	-	-
PS8	-	-	-	-	-	-	-
PS9	-	-	-	-	-	-	-
PS10	-	-	-	-	-	-	-

Table S15. Pore solution addition to NS-5. Values that could not be obtained with DLS were omitted.

Sample	pH	Size (d.nm)	SD	PdI	SD	ZP (mV)	SD
PS0	8.5	-	-	>0.7	-	−53.6	2.3
PS1	9	-	-	>0.7	-	−11.8	1.1
PS2	9	-	-	>0.7	-	−6.3	1.0
PS3	9.5	-	-	-	-	-	-
PS4	10	-	-	-	-	-	-
PS5	-	-	-	-	-	-	-
PS6	-	-	-	-	-	-	-
PS7	-	-	-	-	-	-	-
PS8	-	-	-	-	-	-	-
PS9	-	-	-	-	-	-	-
PS10	-	-	-	-	-	-	-

Table S16. Pore solution addition to NS-20a. Values that could not be obtained with DLS were omitted.

Sample	pH	Size (d.nm)	SD	PdI	SD	ZP (mV)	SD
PS0	4	-	-	>0.7	-	−24.2	1.1
PS1	7	63.3	1.3	0.20	0.01	−7.6	8.8
PS2	8	77.5	0.6	0.29	0.01	2.5	9.4
PS3	9	-	-	>0.7	-	0.1	3.6
PS4	10	-	-	>0.7	-	8.5	2.8
PS5	-	-	-	-	-	-	-
PS6	-	-	-	-	-	-	-
PS7	-	-	-	-	-	-	-
PS8	-	-	-	-	-	-	-
PS9	-	-	-	-	-	-	-
PS10	-	-	-	-	-	-	-

Table S17. Material properties for cryoSEM.

Oxide	CaO	SiO_2	Al_2O_3	Fe_2O_3	SO_3	MgO	Na_2O	K_2O	L.O.I.	ZrO_2+HfO_2	P_2O_5
Cement wt %	68.97	24.48	2.08	0.35	2.08	0.61	0.16	0.04	0.57	-	-
SF wt %	0.02	92	0.3	0.4	-	-	0.01	0.01	-	5	0.3

Table S18. pH adjustment of NS-20b using 0.1 M HCl and 0.1 M KOH.

pH	Size (d.nm)	SD	PdI	SD	ZP (mV)	SD
1.5	29.7	0.2	0.05	0.01	−10.0	3.7
2	30.0	0.0	0.07	0.01	−4.9	0.3
2	29.8	0.1	0.07	0.01	−7.7	0.8
3.5	30.5	0.1	0.06	0.00	−9.7	0.5
5.5	28.8	0.1	0.08	0.02	−3.9	0.8
6.5	26.9	0.1	0.09	0.00	−28.2	0.4
7	29.6	0.1	0.06	0.01	−13.5	0.1
8	24.0	0.1	0.14	0.01	−13.9	0.7
8	23.0	0.4	0.16	0.00	−14.1	0.7
8	26.5	0.3	0.13	0.01	−41.2	0.4
9	26.7	0.2	0.13	0.01	−43.3	3.3
9.5	27.7	0.1	0.10	0.02	−42.4	1.8
10	28.1	0.1	0.10	0.01	−34.4	1.6
10	28.0	0.2	0.11	0.01	−36.3	1.3
10.5	28.7	0.1	0.08	0.01	−37.2	0.1
11	29.0	0.2	0.09	0.01	−37.3	0.7
11	29.2	0.0	0.08	0.00	−35.9	1.7
12	29.7	0.1	0.09	0.01	−40.5	0.8
12	29.2	0.1	0.09	0.01	−35.6	1.5
12.5	29.7	0.1	0.08	0.01	−37.4	1.0

Table S19. pH adjustment of NS-75 using 0.1 M HCl and 0.1 M KOH.

pH	Size (d.nm)	SD	PdI	SD	ZP (mV)	SD
2	90.2	0.4	0.01	0.01	−8.8	0.4
2	88.8	1.0	0.02	0.01	−4.0	0.3
2.5	90.0	0.2	0.02	0.01	−16.3	1.0
2.5	89.2	0.4	0.03	0.00	−13.4	0.5
3	89.4	0.8	0.02	0.01	−6.4	0.7
3	88.9	0.7	0.05	0.02	−7.2	0.3
4.5	89.0	1.0	0.04	0.02	−8.3	0.8
7	96.2	1.1	0.07	0.00	−44.1	1.3
8.5	93.0	0.2	0.06	0.01	−50.7	1.0
9.5	94.2	1.2	0.03	0.01	−50.5	0.6
9.5	93.2	0.7	0.03	0.02	−54.4	3.8
9.5	92.4	1.3	0.04	0.01	−56.7	1.6
10.5	93.5	2.2	0.02	0.02	−54.5	1.9
12.5	94.6	1.3	0.03	0.02	−40.6	1.4

Table S20. pH adjustment of NS-5 using 0.1 M HCl and 0.1 M KOH.

pH	Size (d.nm)	SD	PdI	SD	ZP (mV)	SD
2	14.8	0.0	0.14	0.01	−1.5	0.2
2	14.8	0.1	0.12	0.00	−1.6	0.3
4.5	14.8	0.1	0.12	0.01	−13.1	0.3
6	14.8	0.1	0.13	0.01	−8.4	0.5
6.5	13.4	0.2	0.16	0.02	−24.5	0.9
7.5	11.8	0.1	0.24	0.01	−1.1	0.1
8	10.4	0.1	0.31	0.01	−14.0	1.0
8.5	10.0	0.1	0.32	0.02	−15.4	0.8
8.5	11.0	0.1	0.42	0.00	−38.8	1.3
8.5	10.1	0.1	0.36	0.00	−52.0	4.4
8.5	13.9	0.2	0.50	0.01	−51.0	2.3
8.5	14.0	0.2	0.51	0.01	−50.4	2.0
9	10.9	0.2	0.41	0.01	−46.9	1.2
9	11.9	0.5	0.45	0.02	−41.8	2.3
9.5	11.3	0.1	0.35	0.01	−37.7	1.4
9.5	11.0	0.1	0.42	0.01	−39.5	2.3
10	11.5	0.2	0.30	0.01	−37.6	0.8
10	15.5	1.3	0.40	0.03	−36.2	1.8
11	13.2	0.4	0.24	0.01	−35.2	1.1

Table S21. pH adjustment of NS-20a using 0.1 M HCl and 0.1 M KOH.

pH	Size (d.nm)	SD	PdI	SD	ZP (mV)	SD
2	31.3	0.1	0.12	0.01	−3.1	0.4
2	31.5	0.3	0.13	0.01	−9.1	0.4
2	30.6	0.3	0.13	0.01	−10.1	0.8
3	30.6	0.2	0.14	0.00	−11.7	0.6
3	29.3	0.2	0.16	0.01	−4.4	0.2
3.5	32.4	0.4	0.15	0.02	−13.4	0.3
5.5	22.1	0.1	0.26	0.00	−5.4	0.8
6	15.9	0.0	0.70	0.00	−9.4	0.7
8.5	41.9	1.8	0.27	0.01	−30.7	0.5
9	40.3	1.7	0.24	0.00	−32.5	0.4
10	39.3	1.5	0.18	0.01	−32.6	1.6
11	39.8	1.1	0.17	0.01	−30.5	1.3
12	40.5	1.3	0.15	0.01	−30.9	1.7
13	40.2	1.1	0.14	0.01	−33.1	1.6
